# The *in vitro* Production Potentialities of Secondary Toxic Metabolites by the Fungal Factory *Fusarium verticillioides* Is, Fortunately, Largely Underestimated in Fields: Pioneering Study on Fumonisins

**DOI:** 10.3389/fmicb.2020.562754

**Published:** 2020-10-30

**Authors:** Pascale Marie Aimée Dozolme, Serge Maria Moukha

**Affiliations:** Centre de Recherche Cardio-Thoracique de Bordeaux, INSERM U1045/INRAE, Plateforme Technologique d’Innovation Biomédicale (PTIB), Hôpital Xavier Arnozan, Université de Bordeaux, Bordeaux, France

**Keywords:** fungal factory, secondary metabolite production, mycotoxin, biochemical warfare, *Fusarium verticillioides*, fumonisin, acute cytotoxic firing, ontological genetic program

## Abstract

This study presents fungi infrequently viewed as fungal factories for secondary metabolite production resources such as mycotoxins in *Ascomycota*. Additionally, we demonstrated that biochemical warfare of *Fusarium verticillioides* factory against animal cells is not only due to mycotoxins such as fumonisins, but acute cytotoxic firing is based on different excreted secondary metabolite series, potentially leading to animal and human diseases. In this study, fumonisins, which can be followed by *in situ* localization, quantification, or expression of the key gene implicated in their synthesis, are used to understand secondary metabolite production by this fungus. It is known that *F. verticillioides* produces mycotoxins such as fumonisins on cereals, but until now, there is no evidence demonstrating a method to totally block fumonisin production on feed and food. In this paper, we explained, what was never clearly established before, that fumonisin production depends on two bottlenecks. The fumonisin synthesis and secretion in fungal articles of the mycelium are medium-independent and follow the fungal cell cycle developmental program (ontogenesis). Conversely, the fumonisin excretion into the medium depends on its composition, which also impacts fumonisin biosynthesis level. Using a high-pressure freezing method, we showed that, in non-permissive fumonisin excretion (NPFE) medium, FB1 is sequestered inside extra-vesicles and in the first third of the cell wall next to the plasmalemma, leading to the hypothesis that the fungus develops mechanisms to protect its cytosolic homeostasis against this cytotoxic. In permissive fumonisin excretion (PFE) medium, leading to very high quantities of excreted fumonisins, FB1 localized inside extra-vesicles, crosses the entire cell wall thickness, and then releases into the medium. Our results demonstrated a delayed and lower expression of *Fvpks* gene in mycelium developed on NPFE medium as compared to PFE medium. Conversely, higher amounts of fumonisins were accumulated in NPFE-grown mycelium than in PFE-grown mycelium. Thus, our results demonstrated for the first time that we have to take into account that the synthesis and secretion inside the article of secondary metabolites depend on the occurrence of cryptic biochemical specialized articles, differentiated in the mycelium. However, those are not morphologically different from other colonial hyphae.

## Introduction

Mycotoxin is a human and animal health concern due to the daily meal consumption. The common accepted idea is that the biosynthesis of secondary metabolite (SM) in fungi is quantitatively variable and mysterious as to their appearance or onset, and is dependent mainly on metabolism adaptation in response to the environment (Miller, 2001; Reynoso et al., 2002; Cleveland et al., 2003; Hinojo et al., 2006; Folcher et al., 2009; Maiorano et al., 2009). However, this vision has evolved following experiences in the 1990s on fungal metalloenzyme productions, which demonstrated that these SM productions are due to a change in the expression of metabolism resulting from the appearance of differentiated secondary specialized hyphae, although those articles are not morphologically modified (Moukha et al., 1993a,b). These cryptic metabolic or biochemical differentiations of specialized cells/articles from the aging mycelium in colonial mycelium of the fungus appear in contact with the solid substrate or in the mycelium developed in liquid cultures, dependently on the modification of the medium imposed by the thallus developmental growth stage (Laugero et al., 1996; Ha et al., 2001). Accordingly to the trophophase development modifying the culture medium composition (such as temperature, pH, and C/N composition), the expression, secretion, and excretion of the corresponding SM appear after a few hours or a few days, following a written fungal cell cycle developmental program (ontogenesis) and usually when idiophase is reached (Moukha et al., 1993a). Thus, SM excretion depends on fungal ontogenesis program, and on the importance of the specific environmental regulation parameters such as medium (Wösten et al., 1991; Moukha et al., 1993a,b; Asther et al., 1998). How or when the process should happen is not yet understood, neither at the fungal physiology nor at the molecular levels.

This appearance of secondary hyphae seems to be a general fungal developmental phenomenon in *Basidiomycota* and *Ascomycota* (Wösten et al., 1991; Moukha et al., 1993a,b). These SM excretions do not correspond to the spontaneous arrival of an environmental enemy organism. Fungi excrete preventive toxics for competition with other organisms for medium, in order to put a boundary between the fungus and the pressure of other invasive microorganisms (bacteria or other fungi), while the fungus itself is installed in its niche and intends to stay there (Currie et al., 1999; Fukuda et al., 2005; McMaster University, 2019).

Any current research tracks do not take into account the intrinsic ontogenesis aspect, especially in the field of mycotoxins and *Ascomycota*. Using the *Fusarium verticillioides,* which can be an *in vitro* cultured**/**fumonisins model, the objective of our pioneer work is to present an essential fundamental approach in order to define specific rules in the role of secondary metabolism and production of SM, essential for “biochemical war” in preserving species. *F. verticillioides* (Sacc.) Nirenberg [synonym *F. moniliforme* (Sheldon), teleomorph *Gibberella moniliformis* Wineland] is a fungus, naturally present in the fields, that causes stalk rot and ear rot in cereals such as maize and rice, in which it has been discovered as *Gibberella fujikuroi* species complex (Leslie, 1996; Nirenberg and O’Donnell, 1998; Logrieco et al., 2002; Hinojo et al., 2006; Folcher et al., 2009). This telluric fungus is also found on/in plant residues such in lateral roots abandoned in fields after harvest (White, 1999; Oren et al., 2003). Fungal material such as mycelium and spores can disseminate in the air, but during fungal development, the most drastically cytotoxic damages for animals or human are due to SM produced by the fungus, such as different mycotoxins, those can cause up to 50% yield loss in cereals (Nganje et al., 2004).

Interest in *F. verticillioides* is intense because this fungus can produce several SM, especially fumonisins, in maize grains used for animal feed and human food (Marasas et al., 2001; Butchko et al., 2003; Proctor et al., 2003). While the mycotoxins exert a deleterious pressure on the health of animals and humans, it seems that these are more side effects than a direct adaptive target. In *F. verticillioides*, fumonisin production is under *fum* gene cluster expression control (Proctor et al., 2003, 2006, 2008; Stepien et al., 2011). The presence of *Fusarium* in food does not automatically mean the presence of mycotoxins, whereas the absence of this fungus does not say that the commodity is mycotoxin-free, since toxins persist after mold disappearance (Bullerman, 1979). Inversely, in case of other SM such as antibiotics (penicillin, for example, Turner, 1971; Turner and Aldridge, 1983; Cole and Schweikert, 2003), benefits for human health are higher than deleterious effects. It is one of the reasons why many of these SMs that could possess toxicity activity in the environment are, therefore, potential candidates for medicinal technological or phytosanitary purposes from an anthropic point of view.

More than 28 fumonisins have been isolated and grouped (Rheeder et al., 2002). The most important and well-studied fumonisin B1 (FB1) represents an environmental toxic risk factor in human populations and mice experimentations (Constable et al., 2017). FB1 is implicated in lipid peroxidation and inhibition of protein and DNA synthesis (Abado-Becognee et al., 1998a) and possesses epigenetic properties (Mobio et al., 2000; Kouadio et al., 2005). Fumonisins also play an essential role in teratogen-induced malformations affecting animal, insect, and human health (Kellerman et al., 1990; Colvin and Harrison, 1992; Ledoux et al., 1992; Gelderblom et al., 1993; Abado-Becognee et al., 1998b; Mobio et al., 2003; Kouadio et al., 2005, 2007; Gelineau-van Waes, 2010; Gelderblom and Marasas, 2011; Haschek and Voss, 2013; Smith et al., 2018). Moreover, fumonisins are long-term disease effectors by activating inflammatory cytokines and inducible NO, leading to alter redox homeostasis and oxidative stress. Fumonisins also disrupt sphingolipid biosynthesis (Merrill et al., 1993; van der Westhuizen et al., 1998), inhibit dihydroceramide (Cer) synthase and enhance free S1P (=sphinganine-1-phosphate & sphingosine-1-phosphate), which control cell proliferation and death, and autophagy (Taniguchi et al., 2012; Dellafiora et al., 2018). Thus, fumonisins represent the molecular toxicity key, modifying the “Sphingo-Lipid rheostat” (Verderio et al., 2018). Little is known on the possible role of these fumonisins in plants. FB1 could decrease plasmalemma fluidity (Nora et al., 2020) and produces chloroplast integrity loss (Scala et al., 2013; Xing et al., 2013; Santiago et al., 2020).

In this paper, we demonstrated for the first time *in vitro* regulation modes by which SMs (based on fumonisins) are first synthesized and sequestered inside the mycelium, or alternatively excreted into the culture medium. Thus, we developed permissive fumonisin excretion (PFE) medium to obtain high excreted fumonisin quantities, and non-permissive (= retention) fumonisin excretion (NPFE) medium. Then, we proceeded for FB1 localization in fungal articles, which were developed in these permissive or non-permissive media, to give evidence for *in vitro* fumonisin production. In addition, by coupling fungal extracts from chemical extractions or fungal culture supernatants with a cellular cytotoxic assay [effect-directed assay (EDA)], we attempted to determine the potential existence of other cytotoxic compounds (SM), different from FB1, which are also excreted in the medium or sequestered in the mycelium.

## Materials and Methods

### *In vitro* Culture Conditions for *F. verticillioides* Production of SMs, Illustrated by Fumonisin Mycotoxin Mass Productions

#### Strain, Media, and Samples

*Strain 7600* (*FRC M3125 = NRRL* 20956) *F. verticillioides* from ARS Culture Collection (1815 N. University Street Peoria, IL 61604) is maintained on PDA Petri dishes until experimentation at 4°C during 6 months of storage at the maximum. Agar covered with growing mycelium 7 days after inoculation (d.a.i.) was spotted (75 mm^3^). Ten spots were shown in 50 ml of CMC [0.1% (w/v) NH_4_NO_2_, 0.1% (w/v) KH_2_PO_4_, 0.05% (w/v) MgSO_4_, 6H_2_O, and 1.5% (w/v) sodium carboxy methyl cellulose] medium. Five d.a.i., “spores” were separated from mycelium and agar was removed by filtration on the cheesecloth. Using Neubauer cell, 10^5^ “spores” were inoculated per milliliter of NPFE [5% (w/v) glucose, 0.1% (w/v) Yeast Extract, and 0.1% (w/v) peptone] or PFE [4% glucose (w/v), 1% (w/v) lyophilized maize water extract, 0.1% (w/v) Yeast Extract, and 1% (w/v) glycine] liquid media (50 ml) or on corresponding solid media [1.4% (w/v) agar covered with PVDF membrane]. Solid cultures were maintained static whereas liquid cultures were continuously stirred at 220 rpm. Fungal structures were recovered by two centrifugations (150,000 *g*.min). After microscopic control, supernatants were stored at −20°C until fumonisin quantification. To eliminate the residues adsorbed on the cell wall, inoculum (5 × 10^6^ “spores”), fungal pellets, and mycelia from PVDF membranes were washed three times in 10% (w/v) glucose in water and then recovered by centrifugation (15,000 *g*.min). After lyophilization of fungal material, dried mycelium was aliquoted precisely in batches of 10 mg before being stored in liquid nitrogen until use.

The following parameters were evaluated: *F. verticillioides* growth kinetic by estimating mycelium dry weight (MDW)/ml PFE and NPFE liquid (1.5, 3, 5, 7, and 10 d.a.i) and solid (10 d.a.i.) media, relative *Fvpks/a-Fvtef* subunit expression from dried mycelium obtained from liquid (3, 7, and 10 d.a.i) or from solid (10 d.a.i) PFE and NPFE media, and fumonisin quantification in the media (FB1, FB2, FB3, and HFB1) or in the dried weight mycelia (FB1, FB2, and FB3) after culture in liquid (1.5, 3, 5, 7, and 10 d.a.i) or on solid (FB1, FB2, and FB3, 10 d.a.i) PFE and NPFE media.

Fumonisins were quantified from 7 ml of supernatant liquid (FB1, FB2, FB3, and HFB1, 1.5, 3, 5, 7, and 10 d.a.i) or 7 ml of lyophilized solid (FB1, FB2, and FB3, 10 d.a.i) PFE and NPFE media. FB1, FB2, FB3, and HFB1 were also quantified from inoculum (5 × 10^6^ “spores”) and from 7 ml of 5-day-old CMC-inoculated medium.

Triplicates, for all time points of each condition, were carried out using three independent experiments (*n* = 9).

#### Fumonisin Synthesis Measurement by Relative *Fvpks* Activity

The relative expression of the key *fum* cluster gene *Fvpks* (FUM1 = FUM5, FVEG_00316)/the housekeeping gene α-*Fvtef* subunit (FVEG_02381) was determined using real-time PCR conditions and the 2^−ΔCT^ method (Ish-Shalom and Lichter, 2010; Kuznetsova et al., 2010).

Total RNAs were extracted from 10 mg of dry weight mycelium (MDW) using Trizol reagent (Gibco BDL, Life Technologies) as described in the manufacturer’s instructions, then treated with DNAse I (Sigma Aldrich). Using oligo-dT columns (CliniSciences, Nanterre-France), mRNAs were purified from total RNA. The quality and integrity of mRNA were confirmed by denaturing agarose gel electrophoresis (data not shown), monitored using Nanodrop technology, and also confirmed by Agilent technology (Otterbein et al., 2000; Seddas-Dozolme et al., 2009). One gram of mRNA was reverse-transcribed to obtain the cDNA, using M-MLV reverse transcriptase (Gibco BRL).

The following primers were used:

- For the *Fvpks* gene expression, a fragment of 628 bp was amplified at 60°C using *Fvpks*-forw (5'-GATGCTCTTGGAAGTGGCCTACG-3') and *Fvpks*-rev (5'-CCTGGACCCCAAGGACACTTGT-3') primers (Proctor et al., 2004);
- For the α-*Fvtef* subunit housekeeping gene expression, a fragment of 629 bp was amplified at 60°C using α-*Fvtef* subunit-forw (5'-ATGGGTAAGGARGACAAGAC-3') and α-*Fvtef* subunit-rev (5'-AACATGATRACTGGTACSTCC-3') primers (Lee et al., 2010).

Real-time PCR: all amplifications and detections were carried out using CFX Maestro software coupled to the CFX system (Bio-Rad) using 2x SsoAdvanced™ Universal SYBR® Green Supermix (Bio-Rad), 100 ng cDNA from each sample, and 250 pM from each primer (Forw and Rev) in each 96-well plate. The reactions were cycled 35 times according to the following parameters: 95°C for 30 s, 60°C for 30 s, and 72°C for 1 min, with an initial cycle of 95°C for 10 min. At each cycle, an accumulation of PCR products was detected by monitoring the increase in fluorescence of SYBR Green binding to dsDNA. A non-template control and a no-primer control (negative controls) were run with each assay, and all determinations were performed at least in triplicate to achieve reproducibility.

Analysis of PCR data: the software determines the cycle number, the “cycle threshold” (C_T_), and thus the number of templates (mRNAs) present in the reactions. The results were expressed as the relative expression of *Fvpks* normalized to the α-*Fvtef* subunit, which is the housekeeping gene, always expressed constitutively whatever the stage of development of the mycelium considered.

For each fungal culture, three independent RNA extractions and a real-time PCR triplicate were performed. Means ± standard deviation (SD) and CV% were calculated from *n* = 9 × 3 (27).

#### Quantification of Fumonisin Excreted by or Stored in *Fusarium verticillioides*

Fumonisins were extracted from filtrates by using an adaptation of Shephard et al. (1990) protocol. Filtrates were obtained from 7-ml liquid-culture supernatants, from 1 g of lyophilized fungal material or from lyophilized solid media (corresponding to 7 ml of fresh solid media). In those cases, lyophilized material was rehydrated (20 min, 20°C) in 7 ml of methanol/water (70%/30%; v/v) and then centrifuged (55,000 *g*.min), and supernatants were kept for fumonisin purification. Briefly, filtrate was adjusted to pH 7.0 and enriched fumonisin fraction was eluted by using Bond Elut Strong Anion Exchange (SAX) column system (Agilent) and 1% (v/v) acetic acid in methanol as described by the manufacturer. After evaporation to dryness under a nitrogen stream, dry pellets were stored at −20°C until fumonisin quantification (Bailly et al., 2005; Tardieu et al., 2008).

Dried samples were dissolved in 200 μl of methanol before high-performance liquid chromatography analysis with fluorescence detection (HPLC-FLD) according to Tardieu et al. (2008). Fumonisins were derivatized to be seen in fluorescence. Twenty-five microliters of methanol extract (diluted in methanol when needed to obtain quantity in relation to the calibration curve) were added to 25 μl of borate buffer pH 8.6, 25 μl of water, and 25 μl of OPA reagent (5 mg of O-phthaldialdehyde, 1.25 ml of methanol, and 2.5 μl of *β*-mercaptoethanol). One minute later, 20 μl of this mixture was injected on the chromatographic system composed of an M2200 pump (Bischoff, Leonberg, Germany) connected to a Prontosil C18 column (Bischoff Chromatography, Leonberg, Germany) with a porosity of 5 μm and a size 250 × 4.6 mm. The latter was connected to a fluorescence detector RF 10A XL (Shimadzu, Kyoto, Japan) and a PIC3 acquisition system (ICS, Toulouse, France). The mobile phase was composed of methanol/0.1 M phosphate buffer pH 3.35 (75/25, v/v). The flow rate was 1 ml/min. The excitation and emission wavelengths were respectively 335 nm and 440 nm. The quantities of FB1, FB2, FB3, and HFB1 were determined by linear regression measuring the peak area and comparing it to a standard calibration curve obtained with ranges of standards (Novakits, France) of known concentrations. For each culture condition, three independent cultures and triplicates of fumonisin-enriched methanol fraction were obtained. Results (mean, SD, and CV%) were calculated for three independent HPLC injections for each sample (*n* = 9 × 3).

Main results obtained demonstrated that the quantification procedure is linear, repeatable (CV = 4%), and reproducible (CV = 4.7%). The mean of HPLC-FLD recovery was 98%. These results are in agreement with the AFNOR EN13585 standard.

### *Fusarium verticillioides* Structure and *in situ* Localization Using FB1 Immunodetection Analysis Using Transmission Electron Microscopy (FB1-IDEM) After High-Pressure Freezing

Structure analysis of *F. verticillioides* articles that produced SM was carried out with mycelium developed on solid media in order to focus samples on differentiated articles, which was too difficult using mycelium developed in liquid media. Moreover, using solid media, excreted FB1 could be immunodetected because of accumulation due to PVDF membrane. This immunolabeling would not have been possible using liquid cultures due to the different washings in procedures.

#### *Fusarium verticillioides* Structure Analysis Using TEM

Fungal material was produced on solid PFE or NPFE media (PVDF membrane) in Petri dishes. The high-pressure freezing method was adapted from Marais et al. (2015) with the following modifications: 10 punched samples of 2 mm^3^ each were high-pressure frozen with a Leica EMPACT system.^1^ They were washed three times and then submerged in PFE or NPFE liquid media added with 20% (w/v) BSA in a flat copper carrier and frozen in a high-pressure freezer (EMPACT-1 Leica; Marais et al., 2015). The subsequent cryo-substitution was performed in a Leica AFS2 freeze substitution unit in acetone supplemented with 2% (w/v) osmium tetroxide (OsO_4_), 0.1% (w/v) uranyl acetate, and 0.5% (w/v) glutaraldehyde at −90°C for 84 h. Temperature was then raised up to −50°C at a rate of 3°C/h and then samples were kept at −50°C for 38 h. Samples were subsequently washed three times for 20 min in 100% acetone and then three times for 20 min in 100% ethanol. Samples were embedded by two impregnations for 2 h in 20% (v/v) Lowicryl HM20 in ethanol, then 2 h in 50% (v/v) Lowicryl HM20 in ethanol, and overnight in 75% (v/v) Lowicryl HM20 in ethanol. After three baths of 2 h each in 100% Lowicryl HM20, polymerization was run out under UV for 48 h at −50°C and then 48 h at 20°C under UV. After ultrathin microtone sectioning, sections of 80 nm deep were deposited onto gold-coating grids and samples were observed using a TEM (Philips CM10 80 kV with AMT × 60 camera, Elexience, BIC collaboration). Experiences were conducted in triplicate.

#### *In situ* Localization by FB1-IDEM Detection

Gold-coating grid-deposited fungal sections were treated for 10 min with 20 μl of 0.02 μm filtered 0.1% (w/v) glycine in water at 20°C. Grids were then saturated using 50 μl of 1 mg/ml BSA in TBS (150 mM NaCl and 50 mM Tris-HCl, pH 7.6) containing 2% (v/v) tween 20 for 15 min at 20°C. Immunoreactions were carried out at 20°C for 45 min using 20 μl of anti-FB1 rabbit polyclonal antibodies (Agro-Bio, France) diluted at different concentrations (1/100, 1/200, 1/400, and 1/800) in 0.05% (v/v) Tween 20 in TBS (TBST). Slides quickly washed five times using TBST were then incubated with 20 μl of 5 nm of gold-labeled goat anti-IgG rabbit polyclonal antibodies (= secondary antibodies, Sigma) at different concentrations (1/5, 1/10, 1/20, 1/30, 1/40, 1/50, and 1/100) in TBST for 40 min at 20°C. After five quick washings with TBST, slides were rinsed with 20 μl of 0.02-μm-filtered water and dried at 20°C before TEM observations. Negative control was carried out omitting anti-FB1 rabbit polyclonal antibodies. Experiences were triplicated.

### *Fusarium verticillioides* Fungal Fractionations and Their Acute Cytotoxicity Tests

#### *Fusarium verticillioides* Fungal Fractions

Frozen supernatants (15 ml, 10 d.a.i.) from liquid PFE or NPFE culture media were lyophilized and then resuspended in 7.5 ml of sterile water and directly used for effect-guided cellular testing (PFE-SN and NPFE-SN). Lyophilized fungal materials were water (PFE-MycW and NPFE-MycW) or ethyl acetate (PFE-MycETAC and NPFE-MycETAC) extracted as follows: lyophilized fungal materials were resuspended at 50% (w/v) in water before being extracted overnight at 4°C under agitation. After centrifugation (150,000 *g*.min), supernatants were kept at −80°C, whereas pellets, containing fungal material, were re-extracted in the same conditions. The supernatants coming from the same mycelium were pooled and then lyophilized. The water-extracted mycelia were extracted twice in 50% (w/v) ethyl acetate (ETAC). ETAC supernatants were lyophilized and then stored at −20°C for further experimentations. Lyophilized fungal water extracts (PFE-MycW and NPFE-MycW) were resuspended in water whereas lyophilized ETAC fungal extracts (PFE-MycETAC and NPFE-MycETAC) were resuspended in 10% (v/v) methanol/water.

Extracts [PFE-SN and NPFE-SN, 10% (w/v) PFE-MycW and NPFE-MycW in water, 10% (w/v) PFE-MycETAC and NPFE-MycETAC in 10% methanol/water (v/v)] were diluted at different concentrations in Neuro-2A cell culture medium for acute cytotoxic assays. Each extraction was triplicated from three independent experiments (*n* = 9).

#### Acute Cytotoxic Effects of *Fusarium verticillioides* Biochemical Warfare

The cytotoxicity test is based on the OECD guidance document n°129 (Sérandour et al., 2012). Acute cytotoxic effects of *F. verticillioides* molecules were carried out using EDAs (Brack, 2003; Sérandour et al., 2012) against Neuro-2A cells (mouse neuroblastoma).

Neuro-2A cells were cultured in RPMI medium containing 10% (v/v) FCS, 2% (w/v) L-glutamine, 2% (w/v) glucose, and 1% (w/v) tobramycin in a 5% (v/v) CO_2_ humidified incubator at 37°C.

After supernatant removal, cells were washed with 10 ml of PBS, individualized by 3 ml of 0.05% trypsin (7 min, 37°C). After addition of 7 ml of Neuro-2A cell culture medium, living cells were counted using Neubauer cell. Culture medium was adjusted to obtain 10^5^ cells per milliliter. Neuro-2A cells (100 μl of 10^5^/ml) were deposited in each 96-well plate, incubated 24 h at 37°C in a 5% (v/v) CO_2_ humidified incubator, washed with 100 μl of PBS, and then exposed to different extract concentrations (1/2, 1/100, 1/200, 1/1,000, 1/2,000, 1/10,000, 1/20,000, and 1/100,000), Neuro-2A cell culture medium (100% viability positive control), or vehicle diluted in Neuro-2A cell culture medium (negative controls) that consisted of addition of 5% (v/v) methanol for Myc-ETAC or 5% (v/v) water for MycW fractions (corresponding to the highest concentration used in cytotoxicity assay: dilution 1/2), respectively, or 50% pre-inoculated PFE or NPFE liquid media (highest concentration used in cytotoxicity assay: dilution 1/2). About 0% viability control (negative control for MTT assay) was obtained by omitting cells in wells. After 24 h of incubation, cell viability was estimated by MTT assay (Kouadio et al., 2005). Experiences were made in triplicate. Standardization was established to compare results between plates and to define EC_50_ (=half Effective Concentration) of each extract (*n* = 8 × 3) using statistic estimated by GraphPad Prism (V 6.05).

### Statistical Analysis

Data were expressed as mean ± SD and CV% was calculated from *n* = 9 to *n* = 27, depending on experiences. We used variance analysis and Tukey honestly significant difference tests. Statistical analysis was carried out by using the R software (v2.3.1, 2006) and values of *p* < 0.05 were considered to be significant.

## Results

### *In vitro* Culture Conditions for *Fusarium verticillioides* Mass Production of SMs

#### *Fusarium verticillioides* Mycelium Production in Media

**Figure 1:**
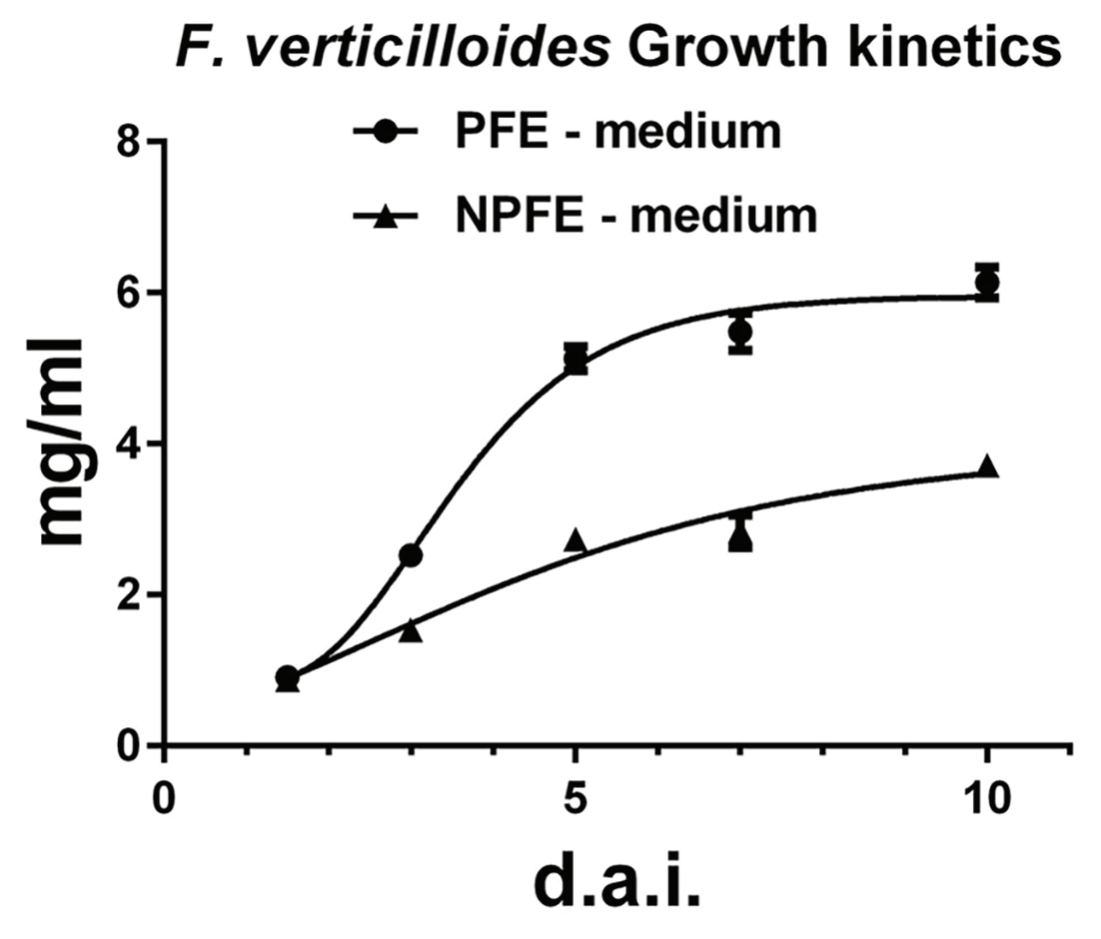
Growth kinetics of *Fusarium verticillioides* cultured in liquid PFE or NPFE media (1.5–10 d.a.i.). Fungal growth rate was estimated by mycelium dry weight obtained after *F. verticillioides* culture in liquid PFE or NPFE media (1.5–10 d.a.i.). Means ± standard deviation (SD) are calculated with *n* = 9, with CV% under 7.39 using GraphPad Prism (V 6.05) software.

Using liquid media, the growth rate, evaluated by the mycelium dry weight (MDW) during 10 days, is 1.65 times higher in the PFE medium (6.13 ± 0.02 mg/ml) than in the NPFE medium (3.71 ± 0.07 mg/ml). Using GraphPad Prism (V 6.05) software, the optimized curve shows that fungal growth reaches a stationary state 5 d.a.i. in PFE medium, whereas only a growth inflection is obtained 10 d.a.i. in NPFE medium (Figure 1). CV% was calculated under 7.39 using *n* = 9.

Using solid media, MDW could only be evaluated 10 d.a.i. when the mycelia covered the PVDF membrane. MDW is 35.2 ± 7.6 mg on PFE medium and 27.3 ± 5.9 mg on NPFE medium using *n* = 9. Statistically, the MDW depending on each solid medium used is not significantly different and CV% reaches 21.59 and 21.61, for PFE and NPFE media, respectively. These CV% are under 30% which is the maximum generally used in biological experiments; nevertheless, we generally use maximum CV% to 20 to keep significant in all our experiments. Under 10 d.a.i., results on MDW could not be included in this study due to CV% >36, which could not be acceptable.

#### Fumonisin Synthesis and Secretion Are Independent of *in vitro* Medium Composition Whereas Fumonisin Excretion Is Medium Dependent

##### Relative *Fvpks* Expression

Data show relative *Fvpks*/α-*Fvtef* subunit expression in mycelia developed in liquid or on solid PFE and NPFE media. Relative *Fvpks*/α-*Fvtef* subunit expression increases during fungal culture in mycelia developed in liquid PFE medium, reaching 3 d.a.i. 368.2 ± 6.8, then 2,867 ± 15 and 4,791 ± 30 7 and 10 d.a.i., respectively.

**Figure 2:**
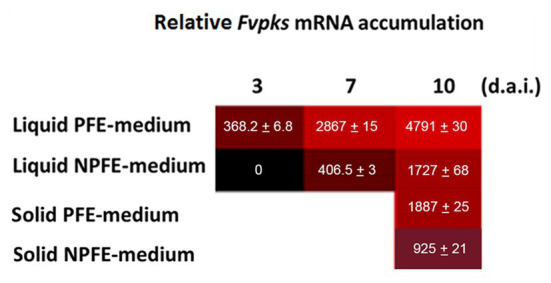
Trevue diagram of relative *Fvpks*/α-*Fvtef*-subunit expression in liquid (PFE, NPFE at 3, 7, and 10 d.a.i.) or solid (PFE, NPFE at 10 d.a.i.) media during *F. verticillioides* culture. Tests were carried out on mycelia grown in PFE or NPFE liquid media (3, 7, and 10 d.a.i.) or on corresponding solid media (10 d.a.i.). *Fvpks* expression was calibrated to this of the housekeeping α-*Fvtef* subunit gene. All values represent means ± SD of three mRNA purifications in triplicates from three independent culture flasks (*n* = 27) with CV% comprised between 0.52 and 3.94, using GraphPad Prism (V 6.05) software.

Remarkably, in mycelia developed in the liquid NPFE medium, while no accumulation of *Fvpks* mRNA is detected 3 d.a.i., relative *Fvpks* mRNA accumulation reaches 406.5 ± 3 7 d.a.i. and 1727 ± 68 10 d.a.i. (Figure 2). Note that relative *Fvpks* expression in liquid PFE medium 3 d.a.i is close to this in liquid NPFE medium 7 d.a.i., implicating the existence of a 4-day delay between both liquid media for relative *Fvpks* expression. Nevertheless, at day 10, relative *Fvpks* expression in liquid NPFE medium is significantly 2.77 times lower than in liquid PFE medium 10 d.a.i.

Using solid media, relative *Fvpks* expression is twice as high in mycelium developed on PFE medium than on the NPFE one 10 d.a.i. (1887 ± 25 with PFE medium compared to 925 ± 21 with NPFE medium; Figure 2).

CV% was estimated between 0.52 and 3.94 with *n* = 27.

Any *Fvpks* mRNA is accumulated in the inoculum (=5 × 10^6^ “spores” developed after 5-day-old CMC medium culture; not illustrated).

##### FB1, FB2, FB3, and HFB1 Accumulation in Liquid PFE and NPFE Media

Fumonisins were extracted from culture media 1.5, 3, 5, 7, and 10 d.a.i. and then quantified.

**Figure 3:**
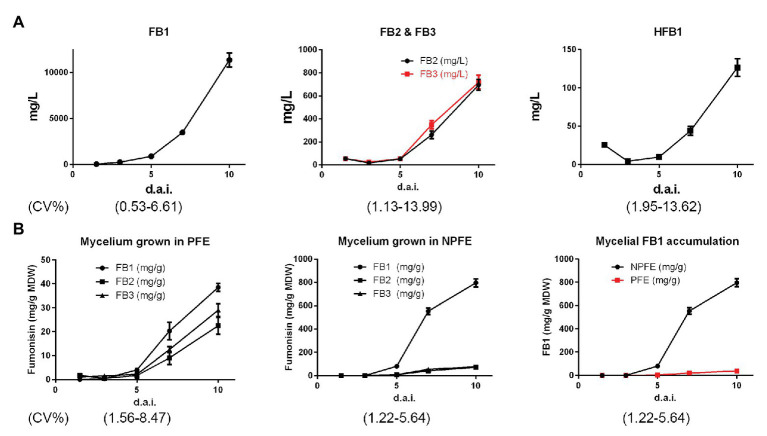
Kinetics of FB1, FB2, FB3, and HFB1 accumulation in PFE liquid media **(A)** and kinetics of FB1, FB2, and FB3 accumulation in mycelium **(B)** during *F. verticillioides* culture from 1.5 to 10 d.a.i. Tests were carried out using PFE culture supernatants **(A)** and PFE-produced mycelia. **(B)** Fumonisins were quantified using standard curves (*n* = 3) for each HPLC quantification. All values represent means ± SD, with CV% determined between 0.53 and 13.99 (*n* = 27) for culture supernatants and between 1.22 and 8.4 for mycelia. In mycelia, quantity is expressed in mg/g of mycelium dry weight (MDW).

FB1, FB2, and FB3 concentrations were nearly similar (about 54 ± 0.6 mg/L) 1.5 d.a.i. and HFB1 quantity represents about half of them (25.7 ± 0.5 mg/L). Except for this time point, FB1 was the major excreted fumonisin in PFE medium whereas FB2, FB3, and HFB1 were also detected at the lower quantity at each time point (Figure 3A). From the kinetic point of view, in the PFE medium, fumonisin quantities increase, reaching the concentrations of 11,360 ± 751.1 mg/L, 695 ± 47 mg/L, 718 ± 53 mg/L, and 126 ± 11.5 mg/L 10 d.a.i. for FB1, FB2, FB3, and HFB1, respectively (Figure 3A). Any fumonisin could be detected in NPFE or in 5-day-old CMC medium culture (not illustrated). CV% was estimated between 0.53 and 13.99 with *n* = 27.

##### FB1, FB2, and FB3 Quantification in Mycelia Grown in PFE or NPFE Liquid Media

At day 10, very few amounts of FB1 > FB3 > FB2 are detected in mycelia developed in PFE medium, reaching 38.5 ± 1.76 > 28.8 ± 2.74 > 22.4 ± 1.6 mg/g MDW, respectively, with FB3 quantity significantly slightly higher than the FB2 one.

Conversely to PFE medium, higher fumonisin amounts were unexpectedly detected in mycelia grown in NPFE medium. In that event, FB1 >>> FB3 > FB2, reaching 796 ± 25.45 >> 65.4 ± 4.18 > 61.4 ± 4.84 mg/g MDW, respectively, with no significant differences between FB2 and FB3 quantities (Figure 3B).

Fumonisin quantities are enhanced in mycelia during culture time but to a lesser extent in PFE liquid medium than in the NPFE one (Figure 3B). In mycelium cultured in PFE liquid medium, FB1 > FB3 > FB2, and at days 3 and 1.5, FB1 = FB3 = FB2. Unexpectedly, in mycelium cultured in NPFE liquid medium, FB1 >>> FB3 = FB2 from day 5 to 10. FB3 = FB2 whatever day considered. Any fumonisin can be detected at days 1.5 and 3 (Figure 3B). CV% was estimated between 1.22 and 8.47 with *n* = 27.

Any fumonisin could be detected in the inoculum (not illustrated).

##### FB1, FB2, and FB3 Accumulation in Media and in Mycelia Developed on Solid PFE and NPFE Media at Day 10

**Figure 4:**
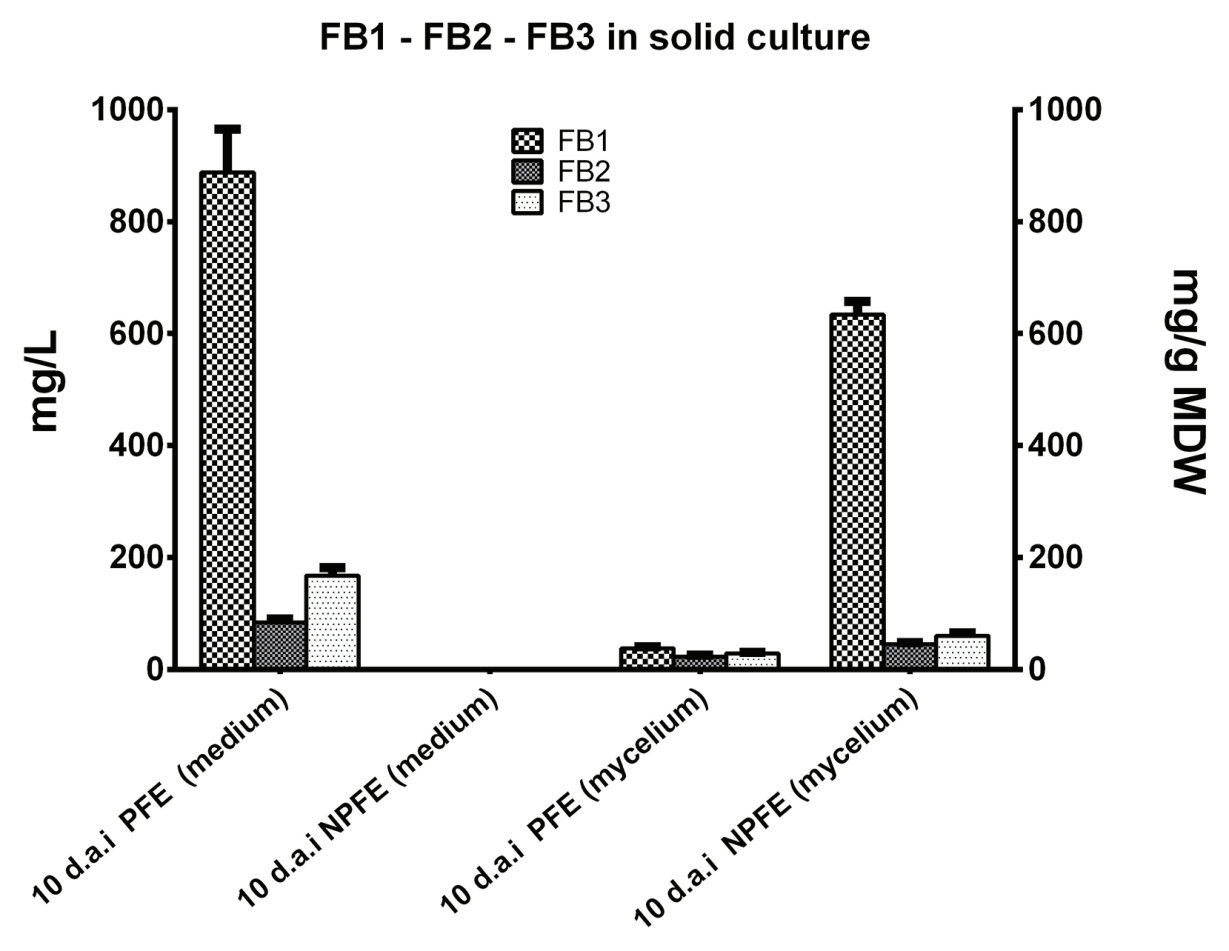
FB1, FB2, and FB3 accumulation in solid NPFE or PFE media and in mycelia developed on the respective media 10 d.a.i. Fumonisin were quantified in semi-solid PFE and NPFE media and in the corresponding dried mycelia, using standard curves. Results are presented in histograms. All values represent means ± SD with CV% comprised between 3.75 and 11.15 (*n* = 27). In mycelia, quantity is expressed in mg/g of mycelium dry weight (MDW).

Amounts of FB1 >> FB3 > FB2 were quantified (887.78 ± 76.93 >> 168.22 ± 14.25 > 84.22 ± 6.14 mg/L) in solid PFE medium 10 d.a.i. (Figure 4), but these quantities are significantly lower than those obtained in the corresponding liquid medium. Any fumonisin is detected in solid NPFE medium (Figure 4).

Fumonisins were quantified in mycelia developed on solid PFE medium: FB1 > FB3 > FB2 (38.15 ± 2.83 > 29.31 ± 1.4 > 22.94 ± 2.56 mg/g MDW; Figure 4). Results go in the same course as those found in mycelium grown in liquid PFE medium and are not significantly different between both PFE media at day 10 (Figure 4).

Unexpectedly, in mycelium grown on solid NPFE medium, fumonisins were quantified. FB1 >> FB3 > FB2 with 634.11 ± 23.8 >> 60.7 ± 4.69 > 45.31 ± 3.07 mg/g MDW, respectively (Figure 4). CV% was estimated between 3.75 and 11.15 with *n* = 27.

Except at day 10, fumonisins could not be estimated in mycelia per gram of MDW due to large variability of MDW estimation (CV% > 36).

### Structure Analysis of *Fusarium verticillioides* Developed on PFE and NPFE Solid Media (10 d.a.i.)

Fungal articles were analyzed using an adaptive embedding and high-pressure freezing protocols (see section Materials and Methods).

**Figure 5:**
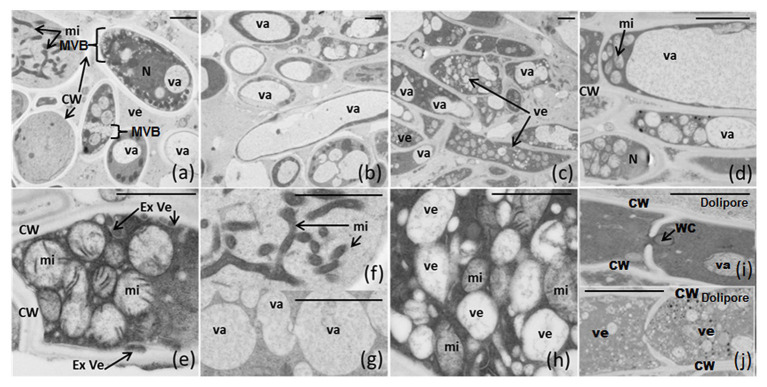
**(A**-**J)** Electron microscopic structure of *F. verticillioides* articles grown on semi-solid NPFE and PFE media after high-pressure-freezing fixation. Sections of 80 nm were observed using Hitachi H7650 TEM (scale bar = 1 μm indicated on each figure). CW, cell wall; Ex Ve, external vesicle; MVB, multi-vesicular body; mi, mitochondria; N, nucleus; va, vacuole; ve, vesicle; WC, Woronin corpus.

Whatever the medium used, some articles contain one or two large vacuoles (Figures 5B,D) and several others possess a high quantity of small vesicles, forming multi-vesicular bodies (MVBs), accumulated in specific places inside the cytosol (surrounding the plasmalemma, or concentrated in the middle of the cytosol between mitochondria; Figures 5A,C). Note the existence of two major networks of linked mitochondria (Figures 5A,E,F) and vesicles (Figure 5G) strongly nested (Figure 5H) in articles. External vesicles (=ectosomes), jammed between plasmalemma and cell wall, are well distinguished (Figure 5E). Dolipore structure and Woronin corpus were also detected (Figures 5I,J), underlining that *F. verticillioides* is an Ascomycota. Vesicles are small enough to go through the septal pore (dolipore) and may use this way to be transported from article to article (Figure 5J).

### FB1 *in situ* Immunodetection Analysis in *Fusarium verticillioides* Developed on PFE and NPFE Solid Media (10 d.a.i.)

Before being used in the FB1-IDEM assay, the specificity of anti-FB1 antibodies was carried out by dot blot using 20 μg of FB1, FB2, FB3, or HFB1 deposited onto a PVDF membrane. Antibody–antigen interaction was revealed by a phosphatase alkaline-labeled goat anti-IgG rabbit polyclonal antibodies and a mixture of 5-Bromo-4-chloro-3-indolyl-phosphate and nitro blue tetrazolium as phosphatase alkaline substrate. The antibodies specifically recognized only FB1 (data not shown).

**Figure 6:**
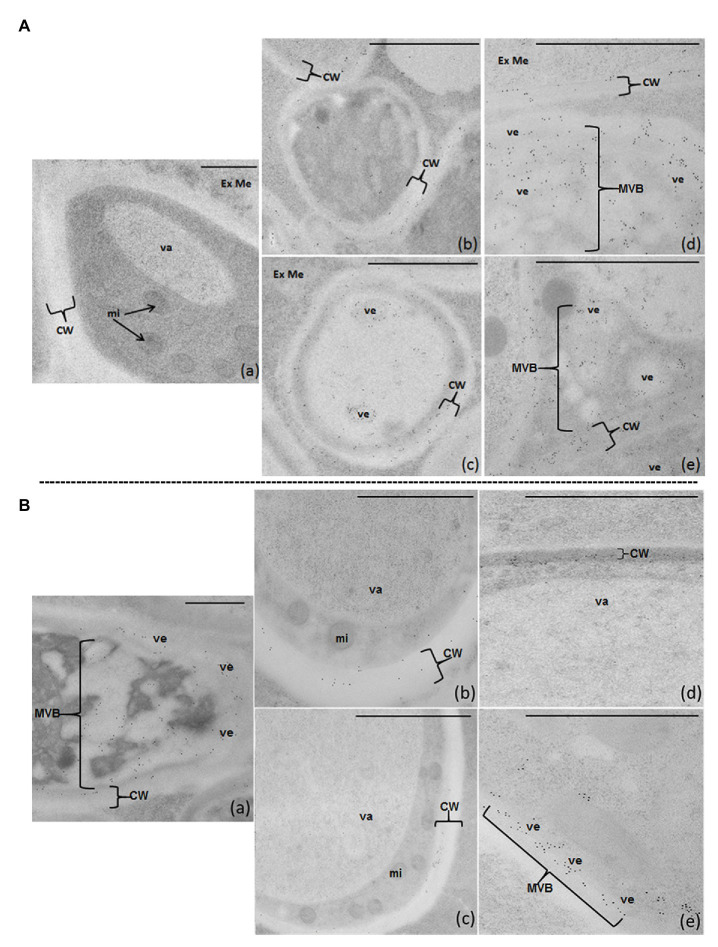
FB1-IDEM localization on 80-nm sections of *F. verticillioides* articles cultured on semi-solid PFE **(A)** or NPFE **(B)** media, after high-pressure-freezing fixation using TEM. Sections are observed using anti-FBI rabbit polyclonal antibodies (1/200) and 5-nm gold labeled goat anti-rabbit IgG polyclonal antibodies (1/30) in TBST. (Aa) represents the negative control in which anti-FB1 antibodies have been omitted (no labeling can be detected). Scale bar is indicated on each figure; a–c = 500 μm, d,e = 100 μm. CW, cell wall; Ex Me, external medium; mi, mitochondria; MVB, multi-vesicular body; va, vacuole; ve, vesicle.

After immunolabeling optimization (as described in M&M), anti-FB1 rabbit polyclonal antibodies were used at 1/200 and 5-nm gold-labeled secondary antibodies at 1/30 in TBST. No labeling could be detected omitting primary antibody, demonstrating anti-FB1 antibody specificity: negative control [Figure 6A (a)].

On PFE medium, FB1 diffuses throughout the cell wall and is excreted outside fungal articles. FB1 is detected inside small vesicles and MVB in the cytosol [Figures 6A (d,e)], crossing the entire thickness of the cell wall [Figures 6A (b-e)] and then excreted into the medium [Figures 6A (b-e)].

Unexpectedly, on the NPFE medium, FB1 is localized inside fungal articles, confined in ectosomes (small vesicles) with some organized as MVB in the cytosol [Figures 6B (a,e)] and in the first third of the cell wall, next to plasmalemma [Figures 6B (a-d)]. FB1 is not observed in the cytosol, mitochondria, nucleus, or in the external medium [Figures 6B (a-e)].

### Acute Cytotoxic Firing of *Fusarium verticillioides* Biochemical Warfare

**Figure 7:**
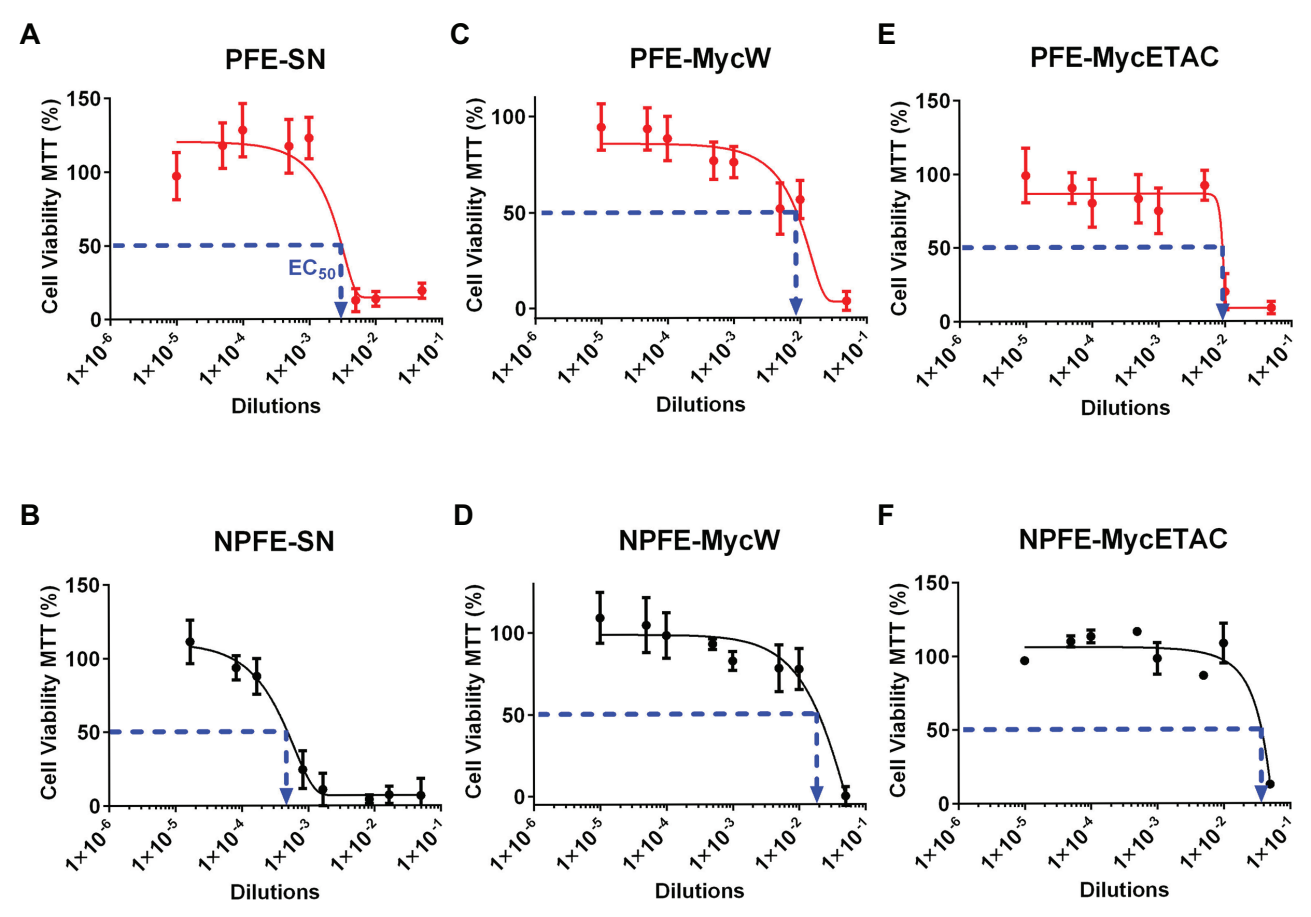
Effect-directed assays of acute cytotoxic biochemical warfare firing of *F. verticillioides*. Supernatants **(A,B)** and water **(C,D)** or ethyl acetate **(E,F)** mycelium extractions from *F verticillioides* cultured in PFE **(A,C,E)** or NPFE **(B,D,F)** liquid media. Acute cytotoxicity was tested during 24 h against Neuro 2A mouse cell line. Results are given as normalization between plates, using three independent experiments (*n* = 8 × 3). IC_50_ is indicated and CV% was calculated under 10.

*Fusarium verticillioides* acute cytotoxicity was determined against Neuro-2A cells by *in vitro* EDA. Fungal culture media cytotoxicity (PFE-SN and NPFE-SN) was higher than those obtained with the corresponding mycelial extractions (compare Figures 7A,B and Figures 7C–F).

NPFE-SN without fumonisin was unforeseen to be more than 10 times more acute cytotoxic than PFE-SN containing FB1: EC_50_(PFE-SN) = 2 × 10^−3^ (Figure 7A) whereas EC_50_(NPFE-SN) = 3.5 × 10^−4^ (Figure 7B). Until dilution of 10^−3^ (PFE-SN) and 10^−4^ (NPFE-SN), viable cells represented 100%.

All fungal extracts from mycelium developed in PFE medium were highly toxic compared to this grown in NPFE medium: [EC_50_(PFE-MycW) = 8.7 × 10^−3^, Figure 7C; EC_50_(PFE-MycETAC) = 9 × 10^−3^, Figure 7E, compared to EC_50_(NPFE-MycW) = 1.9 × 10^−2^, Figure 7D; EC_50_(NPFE-MycETAC) = 3.8 × 10^−2^, Figure 7F]. Using both media, water extractions (Figures 7C,D) were slightly highly cytotoxic than ethyl acetate ones (Figures 7E,F). CV% was calculated under 10, with *n* = 24.

Neither 5% methanol, 5% water, nor 50% pre-inoculated PFE and NPFE liquid media diluted in Neuro-2A cell culture medium affected Neuro-2A viability, which reached 100% (data not shown), suggesting that both pre-inoculated liquid PFE and NPFE media were not toxic against Neuro-2A cells.

### Statistical Analysis

In our studies, all CV% were under 16.33%, except for MDW on solid medium where CV% reaches 21.59 and 21.61. CV% until 30% is usually accepted for biological experimentations (Brack, 2003), but we generally used maximal CV% at 20 as accepted result in our practice.

## Discussion

We have optimized the time course for obtaining high quantities of inoculum (“spores” after 5 days of culture in CMC-medium) to sow media. PFE medium was optimized to achieve a high amount of excreted FB1 into medium and, to a lesser extent, FB3, FB2, and HFB1 excretions. Differences in fumonisin excretion could not be explained by differences in mycelium amount obtained according to media. In NPFE medium, any fumonisin could be detected neither in liquid nor in solid media. Before us, previous works underlined that fumonisin production depends on the fungal strain and media composition (Miller, 2001). However, the authors obtained only a small quantity of fumonisins in liquid media. Proctor et al. (1999) produced 210 mg FB1/L of GYAM after 2 weeks and transformation of *G. fujikuroi* with *Gfpks* multicopy gene. Thiel et al. (1991) obtained 7.1 mg FB1/g of dehydrated double autoclaved kernels in water containing *F. moniliforme*, leading to confusion whether FB1 is in the mycelium or excreted in the medium. Moreover, 280 mg of FB1 + FB2/L malt extract medium was obtained 10 d.a.i. in a jar using *F. moniliforme* (Miller et al., 1994). Solid-state media do not seem to improve FB1 production, and maximum excreted FB1 quantities were obtained with *F. verticillioides* cultured on malt and oatmeal extracts or on PDA (Frisvald et al., 2007). Conversely, FB2 maximum quantity was discharged by *Aspergillus niger* on Czapek-yeast-autolysate-agar medium supplemented with sucrose or NaCl or on rice-corn-steep agar. In these culture conditions, *F. verticillioides* did not excrete fumonisins (Frisvald et al., 2007). Thus, the highest fumonisin quantities, obtained by all these authors in *in vitro* culture 10–14 d.a.i., were obtained in our study only after 3 d.a.i., suggesting that based on media and fungal culture conditions, *F. verticillioides* is a real SM factory (Stone, 2018).

Moreover, in mycelia developed either in liquid or on solid NPFE media, after 10 days of culture, higher fumonisin quantities were obtained in our conditions than those obtained previously *in vitro* by several authors in their medium. Fumonisin quantities in PFE medium found in our experiments represent about 43 times higher amounts of FB1 + FB2 obtained in medium by Miller et al. (1994) and more than 54 times for FB1 quantity obtained by Proctor et al. (1999) in GYAM medium. Just for comparison, for a production of 10 g of fumonisins contained per liter of culture medium as we produce, it would be necessary to harvest and extract more than five tons of infected grains. This represents 10 m^3^ of fumonisin-contaminated maize grains, to obtain this same quantity, if we consider that grain contamination in the field reaches 2000 μg of fumonisins/maize grain kg (which is currently regarded as a rather high level of contamination of harvested grains, World Health Organization, 2018). In the fields, we are undoubtedly far away from the optimal production capacity of the fungus. The quantity of fumonisins quantified in the field represents, fortunately, only an escape of the fungal expression potential in a most favorable environment.

We demonstrate at the initial time that fumonisins are always synthesized and secreted inside *F. verticillioides* mycelium. Fumonisins are localized in vesicles such as MVB or found in the cell wall. This localization was here independent of the medium composition used herein. In CMC medium, the filamentous form is scarce with the majority of numerous oblong cells, “spores,” used for inoculum. The synthesis or production of fumonisins does not accompany this fungal development as “yeast-like” in CMC medium. Moreover, it appears that fumonisin excretion is medium composition dependent, allowing pull up *Fvfum* cluster expression and consequently fumonisin accumulation in medium. We thus hypothesize that fumonisin synthesis is under the control of the genes that regulate the fungal development, including the coordination to metabolic changes in ontology cycle onset without real morphological change in secondary hyphae, as it was previously demonstrated in *Basidiomycota* (Moukha et al., 1993a,b). Fumonisin excretion into the medium is under the control of environmentally modulated genes, which command specific transcription pathways, triggering the specific protein level and enzyme biosynthesis to ensure adaptation and fungal survival. Our hypothesis is supported by our results on relative *Fvpks* expression, by fumonisin quantification in mycelia grown in PFE or NPFE media, and by FB1-IDEM localization in fungal articles.

Thus, based on the problem of production of mycotoxins (especially of fumonisin B1 since this SM is at the origin of public and animal health problem), we hypothesize a common phenomenon for production of all SMs, and all fungi are concerned whether they belong to *Ascomycota* as well as to *Basidiomycota*. From the development of these multicellular thallophytic organisms, new biochemically differentiated hyphae appear over time. This phenomenon is more visible with the aerial hyphae, because moreover they differ morphologically from surface hyphae due to specific metabolite productions, resulting from the development of their particular ontogenesis, very likely linked to a background ontogenetic program. Among these aerial hyphae, some articles differentiate to produce fruit bodies once the aerial hyphae aggregated, or also to produce asexual sporophores, all necessary for fungal sexual or asexual reproduction. Some of these morphological differentiations are also dependent on environmental parameters, including light or osmoregulation (Kües and Liu, 2000). Hence, all the hyphae constituting the fruit bodies are not biochemically similar, and some will specialize in giving the color of the fruit bodies or conidia for examples. The phenomenon of reproduction and distribution of the sexual or asexual organs is often perceived as random, particularly when it is attempted to reproduce a sexual or asexual complete life cycle of a fungus in the laboratory. This random aspect also occurs in the case of the production of SM, leading to a hyphal cryptic biochemical differentiation of the so-called vegetative surface mycelium. The biochemical differentiation phenomenon of hyphae occurs therefore ubiquitously at the level of all articles of the mycelium, each specializing according to one well-defined genetic program to produce specific SM (Rosin et al., 1985; Moore et al., 2008). Implementation of SM with fungal differentiation is a complex process, and an elaborate surveillance apparatus seems to be required, implicating gene expression orchestration (Moukha et al., 1993a,b).

Using NPFE medium, FB1 is detected inside vesicles and MVB, in ectosomes (ve) or confined in the inner 1/3 of cell wall next to the plasmalemma. Thus, growing on NPFE medium allows one to hypothesize that toxic molecules are trapped in vesicles and MVB in order to certainly protect fungal cytosol homeostasis against potential deleterious/toxic FB1 effects. FB1 trapped in the inner 1/3 of the cell wall shows also that this fumonisin is not authorized to diffuse freely through the entire cell wall, indicating that broadcast blocking is provided by the cell wall physicochemical composition, which may itself depend on cultural environment. Because high quantities of fumonisins are detected inside mycelium, we hypothesize that when fumonisins are detected in cereals without the presence of the fungus, this fact could be due to disappearance of dead mycelia, leaving fumonisins in grains as remaining traces of their dislocated corpses.

Growing in PFE medium authorizes a second hypothesis, in agreement with the first one, when FB1 biosynthesis is abundant. Within the fungal mycelium, FB1 can use cell wall and/or vesicle networks for direct diffusion or to migrate from subapical articles to the apex of hyphae to be further excreted when environmental conditions are optimum. In that case, as suggested by our TEM results, looking at vesicle size, we hypothesize that vesicles could use septal pore (dolipore) way to be transported from articles to subapical articles to the apex of hyphae. In PFE medium, FB1 diffuses from mycelium to medium. However, we cannot specify whether FB1 scatters from each fungal cell article or whether a specific apex diffusion mechanism of sequestering vesicles is used as for *Phanerochaete chrysosporium* peroxidases (Moukha et al., 1993a), *A. niger* α-amylases (Miller et al., 1994), or *Picnoporus cinnabarinus* aromatic compounds and laccase (Asther et al., 1998).

In eukaryotic cells, extracellular vesicles (EVs) generate at the plasmalemma (ectosomes = microparticles/microvesicles) or inside vesicles (= exosomes) that could be organized as MVB (Verderio et al., 2018). It is well known that FB1 modifies the “SphingoLipid rheostat.” Sphingolipids, such as Cer and S1P, facilitate EV biogenesis, promoting vesicle buddings inside MVB and ectosomes (Nora et al., 2020). Our TEM results demonstrate the existence of both types of EV. EV is known as cell-to-cell communication mediators due to their ability to transfer biomolecules among cells and to influence the extracellular microenvironment *via* regulation of critical nutrients (Nora et al., 2020). *F. verticillioides* needs to be considered as a producing ready-to-use SM factory and could be interpreted as an armed soldier, ready to shoot for maintaining its species survival. FB1, stored in EV, suggests its involvement in the cytotoxic biochemical shooting of the fungus, according to the relevant environmental conditions.

*F. verticillioides* cannot be viewed as a strict plant pathogen because it can easily be *in vitro* cultured and SM can relatively be well expressed. Moreover, fumonisins are undoubtedly produced by *in vitro* culture isolates, suggesting that plant–fungus interaction is not required for fungal SM productions. Our results match with previous works, emphasizing that *F. verticillioides* develops a non-close invasive relationship with the plant (Moukha et al., 1993a; Asther et al., 1998; Hinojo et al., 2006). Moreover, *F. verticillioides* does not possess the capacity to form appressoria on plant (Howard, 1997; Tollot et al., 2009). Besides, it seems that there is yet no proof of specific gene dialog between protagonists such as exists in plant/pathogen (Gupta et al., 2015) or endo-symbiosis interactions (Seddas-Dozolme et al., 2009; Kuznetsova et al., 2010; Tisserant et al., 2012). Nevertheless, *F. verticillioides* status as plant pathogen of maize or rice is still in discussion concerning SM productions (Perincherry et al., 2019). Primary contaminated tissues in maize are the ears *via* a stylar canal (Miller, 1919; Duncan and Howard, 2010), leading to consider *F. verticillioides* as a female reproductive apparatus parasite and subsequently a pest of the embryos and the seeds, where the symptoms are concentrated. Whereas major fungal damages are often only localized in the ears, some spot invasions could appear on surrounding leaves (Munkvold and Desjardins, 1997).

Using chemical fractionations, we also demonstrate as a pioneering result that a 24-h exposure with *F. verticillioides* extracts that do not contain FB1 as a major toxin (non-permissive FB1 excretion culture supernatant or permissive mycelium containing low FB1) affects Neuro-2A cells. We hypothesize that *F. verticillioides* synthesizes and excretes other cytotoxic SM against neuronal cells. Thus, it appears that (i) biochemical armaments of this fungal factory against animal cells seem multiple, (ii) this *Ascomycota* develops different biochemical warfare processes to ensure its survival in different environmental conditions, and (iii) that fungal cytotoxic firing is based on different excreted SM series. Our results match with the existence of 39 *Fvpks* clusters in *F. verticillioides* genome (Ma et al., 2010), suggesting that different SM potentially toxic could be produced. Work is underway on different animal/human cell lines to identify these SM and cell type specificity for FB1.

In conclusion, our theory is that productions of *Ascomycota* fumonisins and more generally SM function as a double-locked lock with two identified bottlenecks. (i) The synthesis and secretion of fumonisins or SM inside the articles are under control and closely related to the natural fungal development program, the understanding of which is still in infancy, whereas (ii) fumonisin or SM excretion into the medium is under environment dependence. Discrete changes in cellular biochemical differentiation in articles certainly trigger the high expression of the *Fvpks* gene of the *fum* cluster and the production of exogenous fumonisins, without drastic morphological modification of these specialized articles. Nevertheless, depending on the medium composition, mycotoxins can remain endogenous inside articles where cell wall structure seems to be not permissive to its diffusion. We thus hypothesize that, like mycotoxins, SM productions depend on fungal ontogenesis for each article, leading to SM excretion as an armament to fight for life depending on environmental conditions. Moreover, this phenomenon should be ubiquitous to all fungus belonging to different families, and it could be extrapolated to animal- or vegetal-specific organs to preserve the survival of the species during life constraints. In contrast, all of this opens up the field of SM potentialities in public and animal health, especially for developing prevention strategies or therapeutic methods that medicine could use.

## Data Availability Statement

The raw data supporting the conclusions of this article will be made available by the authors, without undue reservation.

## Author Contributions

All authors listed have made a substantial, direct and intellectual contribution to the work, and approved it for publication.

### Conflict of Interest

The authors declare that the research was conducted in the absence of any commercial or financial relationships that could be construed as a potential conflict of interest.

## Footnotes

^1^http://www.leica-microsystems.com

## Abbreviations

Cer: Ceramide
d.a.i.: Days after inoculation
DW: Dried weight
EV: Extra-vesicle
MDW: Mycelium dry weight
MVB: Multi-vesicular body
NPFE: Non-permissive fumonisin excretion
PFE: Permissive fumonisin excretion
S1P: Sphinganine-1-phosphate and sphingosine-1-phosphate
SM: Secondary metabolite
